# Bayesian Reconstruction Algorithms for Low-Dose Computed Tomography Are Not Yet Suitable in Clinical Context

**DOI:** 10.3390/jimaging9090170

**Published:** 2023-08-23

**Authors:** Inga Kniep, Robin Mieling, Moritz Gerling, Alexander Schlaefer, Axel Heinemann, Benjamin Ondruschka

**Affiliations:** 1 Institute of Legal Medicine, University Medical Center Hamburg-Eppendorf, 22529 Hamburg, Germany; m.gerling@uke.de (M.G.); heinemann@uke.de (A.H.); b.ondruschka@uke.de (B.O.); 2 Institute for Medical Technology and Intelligent Systems, Hamburg University of Technology, 21073 Hamburg, Germany; schlaefer@tuhh.de

**Keywords:** Bayesian deep learning, radiation exposure, sparse-view CT, POTOBIM

## Abstract

Computed tomography (CT) is a widely used examination technique that usually requires a compromise between image quality and radiation exposure. Reconstruction algorithms aim to reduce radiation exposure while maintaining comparable image quality. Recently, unsupervised deep learning methods have been proposed for this purpose. In this study, a promising sparse-view reconstruction method (posterior temperature optimized Bayesian inverse model; POTOBIM) is tested for its clinical applicability. For this study, 17 whole-body CTs of deceased were performed. In addition to POTOBIM, reconstruction was performed using filtered back projection (FBP). An evaluation was conducted by simulating sinograms and comparing the reconstruction with the original CT slice for each case. A quantitative analysis was performed using peak signal-to-noise ratio (PSNR) and structural similarity index measure (SSIM). The quality was assessed visually using a modified Ludewig’s scale. In the qualitative evaluation, POTOBIM was rated worse than the reference images in most cases. A partially equivalent image quality could only be achieved with 80 projections per rotation. Quantitatively, POTOBIM does not seem to benefit from more than 60 projections. Although deep learning methods seem suitable to produce better image quality, the investigated algorithm (POTOBIM) is not yet suitable for clinical routine.

## 1. Introduction

Computed tomography (CT) is one of the most advanced achievements in diagnostic radiology and is now a widely used method, but it has led to increased radiation exposure in the general population [1]. In the early 2000s, there was growing concern about CT due to the higher doses required compared with conventional radiography and possible radiation-induced carcinogenic effects [1,2]. Thus, in the period from 2007 to 2016, the use of CT examinations increased by approximately 45%, with CT accounting for 9% of the procedures used in medicine, corresponding to 67% of the collective effective dose [3]. However, the ALARA (As Low As Reasonably Achievable) principle legally requires that radiation exposure be kept as low as possible, even below regulatory limits [4]. Nevertheless, sufficient image quality is required for CT scans to provide the desired diagnostic value, otherwise they must be omitted.

The high radiation exposure and long acquisition time arise from the large number of X-ray projections acquired at different angles, from which the tomographic slices are computed. Commonly, 1000–2000 projections are acquired per rotation (dense-view) from which the CT scan is reconstructed. 

A sparse-view CT offers an alternative where the image is computed from fewer than 100 projections per rotation [5], but all these efforts must fulfill the requirement of comparable accuracy. Reducing the number of projections reduces radiation exposure and could additionally reduce acquisition times and costs [6]. However, sparse-view CT is accompanied by a loss of image quality and the reduced projections can cause significant artifacts when considering conventional reconstruction algorithms. Therefore, there is a strong desire for alternative reconstruction algorithms that provide a comparable image quality to routine examination CTs but with a reduction in radiation exposure. Besides medical application, sparse and especially incomplete data is also prevalent in industry CTs, where large objects or objects with reduced accessibility limit the number of projections that can be acquired [7]. Analogous to medical CT, the reduced number of projections used can lead to artifacts or to high scanning times when using too many projections [8].

In medical CTs, filtered back projection (FBP) was considered the standard method of diagnostic CT reconstruction until a few years ago. FBP is considered a fast and robust method for generating CT images of reasonable quality. However, significant image noise and artifacts can occur if not enough radiation dose is applied [9,10].

Another reconstruction technique—iterative reconstruction (IR)—which has been further developed in recent years makes it possible to obtain equivalent image quality in terms of image noise at the same dose, with the aim of reducing the radiation dose (preserved image sharpness, lower image noise). In the early years of CT, IR was already used for reconstruction purposes, but was initially displaced by FBP due to increasing data volumes [10]. In the intervening years, however, IR has emerged as a standard CT reconstruction technique [11]. Iterative approaches such as the originally proposed algebraic reconstruction technique (ART) [12] or later refined techniques, e.g., model-based iterative reconstruction [13], can provide superior image quality and are less effected by incomplete data or a reduction in projection angles as in sparse-view CTs [14,15]. Specialized reconstruction algorithms for sparse-view CTs have also been proposed for incomplete data in different industrial settings [16,17].

More recently, deep learning methods have been proposed for a broad field of applications and also for CT reconstruction issues, e.g., automated transform by manifold approximation [18]. For sparse-view reconstruction, the model parameters are learned based on pairs of low-dose and high-dose image pairs in a supervised fashion. However, these models are sensitive to variations in the input data [19] and the reconstruction artifacts can include hallucinations of anatomical features not included in the sinogram [20]. To address these concerns, deep image prior (DIP) has been proposed in which the model is parameterized separately for each reconstruction during unsupervised training [21]. But DIP is vulnerable to overfitting and therefore requires manual intervention in early stopping or model under-parametrization. Consequently, a Bayesian neural network (BNN) approach to DIP has been considered to automate the prevention of overfitting [22,23]. Outperforming previous approaches, Posterior Temperature Optimized Bayesian Inverse Models (POTOBIM) have recently shown the most successful application of Bayesian DIP in the context of sparse-view reconstruction [24]. However, a clinical test of its applicability in routine CT scans has not yet been investigated. 

Therefore, in this work, we evaluate the clinical readiness and limitations of sparse-view reconstruction based on BNNs for post mortem CT evaluation as model of whole- body scans in clinical use. The deciding factor for the viability of the reconstruction is the diagnostic value and not just reduced error metrics. Therefore, we consider the reconstruction of post mortem slices with clinically relevant anatomical structures. In addition to quantitative error metrics, we consider qualitative evaluation by an expert radiologist with years of experience in forensic imaging. We compare the dense-view ground truth with simulated sparse-view reconstructions for both FBP and POTOBIM. 

This investigation shall discover to what extent these newly developed methods produce qualitatively sufficient and comparable images that can also be used to recognize and evaluate important structures and to what extent they can keep up with established methods despite a lower dose. We hypothesize that POTOBIM is superior to FBP and comparable to dense-view ground truth (first hypothesis). 

Furthermore, we want to interpret the results of the quantitative and qualitative evaluation. We hypothesize that the results of the quantitative evaluation represent the qualitative/subjectively perceived quality to a similar degree, i.e., that quantitatively measurable changes are also qualitatively perceptible.

## 2. Material and Methods

### 2.1. Post Mortem Computed Tomography

Philips Incisive Plus 128-slice MDCT (Philips GmbH, Hamburg, Germany) was used for 3 different scans. Whole-body scans with a slice thickness of 1 mm, slice spacing of 0.75 mm, 0.5 pitch, 120 kV voltage and a current of 113–130 mA. Head scans with a slice thickness of 0.8 mm, slice spacing of 0.65 mm, 0.3 pitch, 120 kV voltage and a current of 300 mA. Pelvis-leg scans with a slice thickness of 0.8 mm, slice spacing of 0.65 mm, 0.5 pitch, 120 kV voltage and a current of 110–212 mA.

### 2.2. Data Collection

Post mortem CT data was collected for 17 corpses during a scientific death case evaluation. To create a data set of high variability, CT scans from different anatomical regions with a representative selection of interfaces of tissues of different radiographic density was performed. 

Both sides of the femur and the fifth lumbar vertebra were chosen as examples of bones. The transition from the heart to the lungs and pneumonia as a typical pathology within the lungs were considered as thoracic regions of interest. Total hip endoprostheses and dental crowns were selected as typical medical foreign bodies for their demarcation from surrounding tissues. Another important structure selected was brain tissue with the distinction from subdural hematoma. The spleen, kidney, pancreas and thyroid gland were selected as examples of organs. The rectus abdominis muscle was chosen as an example of musculature. In addition, the subcutaneous adipose tissue and, within the soft tissue, a hematoma (exemplarily at the right forehead) were selected for examination.

### 2.3. Sparse-View Reconstruction

**Figure 1:**
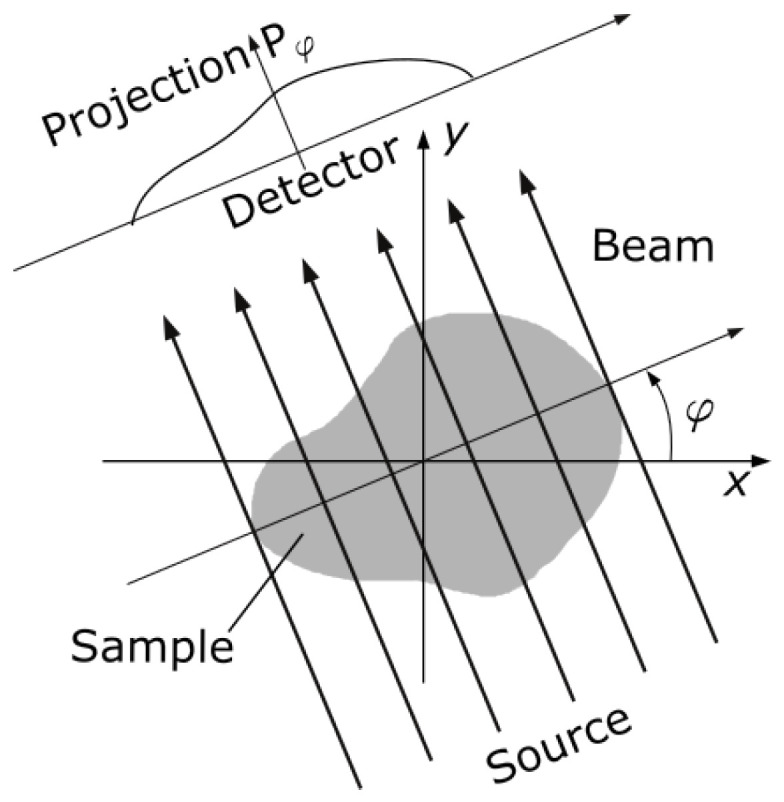
Schematic of parallel beam geometry that was considered to simulate sparse-view sinograms via the forward Radon transform. The projection angle (φ) was varied between 0° and 180° with different, equidistant step sizes to simulate sinograms with 8, 20, 40, 60 and 80 projections per rotation.

Given the reference dense-view reconstructions, corresponding sparse-view data is simulated via the forward Radon transform. We assume a parallel-ray projection geometry and simulate sparse-view sinograms with 8, 20, 40, 60 and 80 projections per rotation. For each sinogram, the simulated projection angles (φ) are uniformly distributed between 0° and 180° (Figure 1). Sparse-view CT slices were then reconstructed from the simulated sinograms using POTOBIM and FBP with a Shepp–Logan filter. For the former, configurations for temperature candidates and iteration cycles were used as suggested by Laves et al. [24] for the task of sparse-view CT reconstruction. 

### 2.4. Quantitative and Qualitative Evaluation

We reconstruct slices from the simulated sparse-view sinograms using POTOBIM. We additionally reconstruct with conventional FBP to visualize the differences. We quantitatively evaluate the reconstructed slices using peak signal-to-noise ratio (PSNR) and structural similarity index measure (SSIM). The PSNR is calculated based on

$$PSNR=20\;{log}_{10}\left(\frac{{MAX}_{f}}{\sqrt{MSE}}\right)$$

With the maximum signal value ${MAX}_{f}$ and the MSE (mean squared error) with respect to the reference image. The SSIM is calculated according to Wang et al. [25]. Based on these findings, a good trade-off between the evaluation metrics and the increased dose was found for 40 projections. Consequently, the comparison by the expert was exemplified conducted for 8, 40 and 80 projections per rotation. These three different categories therefore cover the majority of the range considered as sparse-view CT (<100). Error metrics were evaluated in python using the Scikit-image toolbox [26].

The visual (qualitative) evaluation was performed as a comparison between the reference image (dense-view) and the sparse-view reconstruction with both POTOBIM and FBP. Images were graded according to a visual grading analysis (VGA) according to Ludewig et al. [27]. 

For relative assessment, which was used in this study, one or more reference images were needed. In this case, the quality of the target structure is compared with the corresponding target structure in the reference image. Using a 3, 5, or 7 point scale, the assessment can be categorized semi-quantitatively [27].

In this study, a modified version of Ludewig’s 5 level scale was applied:

- 1—test image clearly better than reference image
- 2—test image slightly better than reference image
- 3—test image equal to reference image
- 4—test image slightly worse than reference image
- 5—test image clearly worse than reference image

It needs to be considered that better images at reconstruction (grades 1 or 2) with reduced sinogram are not to be expected. Qualitative evaluation by the radiologist expert was conducted by displaying the two images side-by-side for each combination of anatomical structure, reconstruction algorithm and the number of projection angles. 

### 2.5. Statistical Analysis

Statistical analyses were performed with SPSS (Version 29.0.0.0 (241), IBM Corp. (Armonk, NY, USA). One-Sample Wilcoxon Signed Rank Test was performed for projections at 8×, 40× and 80× (POTOBIM and FBP) in comparison to the reference image. The level of significance was defined as *p* < 0.05.

## 3. Results

### 3.1. Visual Grading

**Figure 2:**
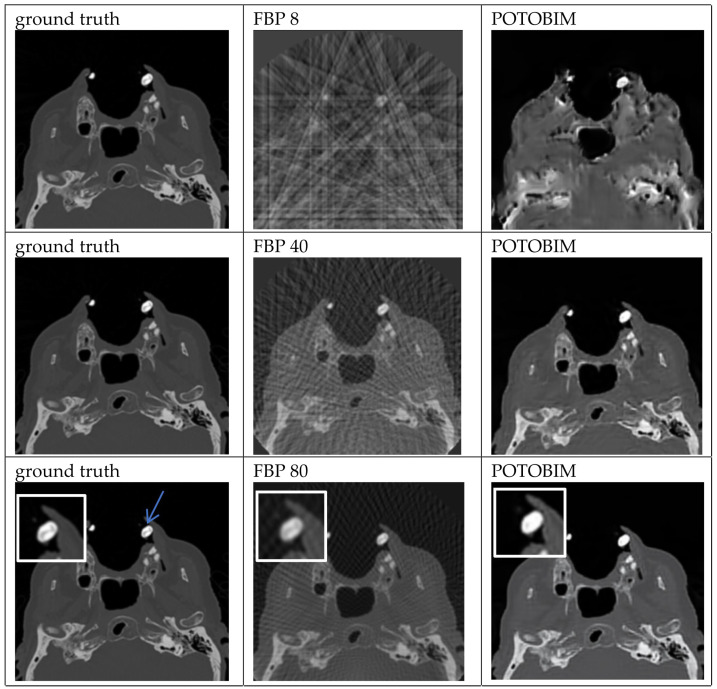
Exemplified images of a dental crown (blue arrow) for reconstructions with 8, 40 and 80 projections used with the FBP (**center**) and POTOBIM (**right**). In comparison, the reference image using all projections (**left**). Detailed view (white box) displaying relevant anatomical region.

**Figure A1:**
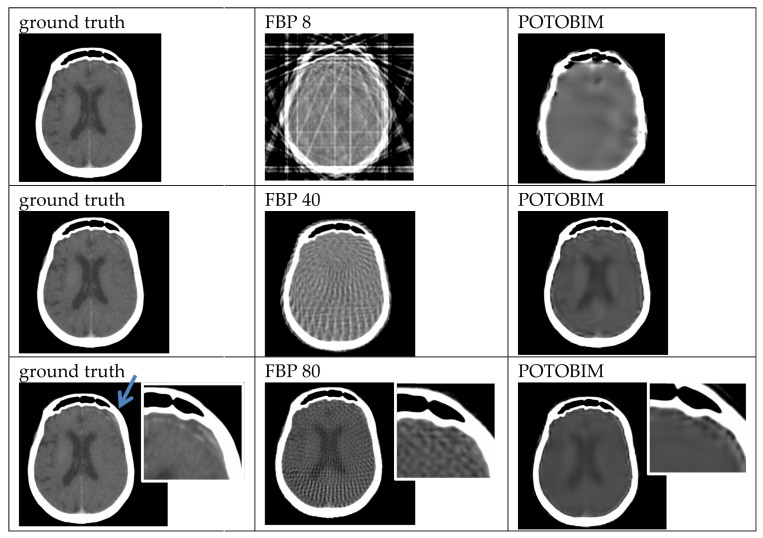
Exemplified images (subdural hematoma left, blue arrow) for reconstruction with 8, 40 and 80 projections used with the FBP (**center**) and POTOBIM (**right**). In comparison, the reference image using all projections (**left**). Detailed view (white box) displaying relevant anatomical region.

**Figure A2:**
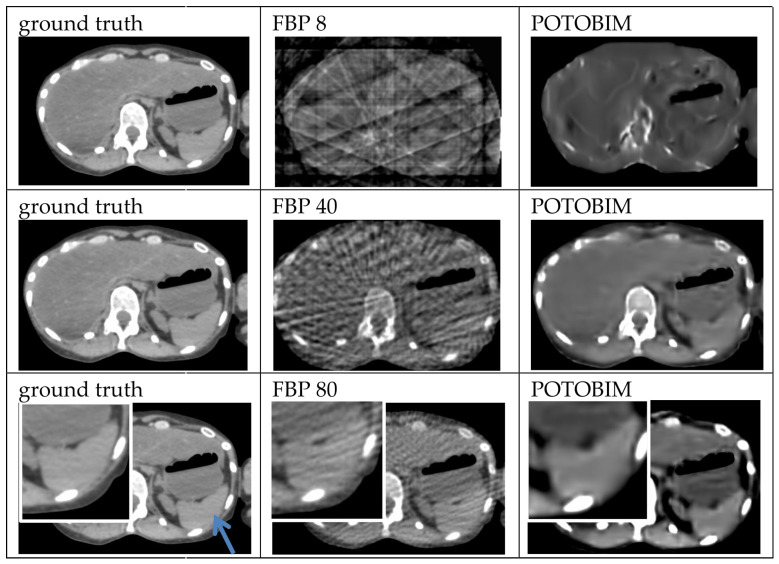
Exemplified images (spleen, blue arrow) for reconstruction with 8, 40 and 80 projections used with the FBP (**center**) and POTOBIM (**right**). In comparison, the reference image using all projections (**left**). Detailed view (white box) displaying relevant anatomical region.

**Figure A3:**
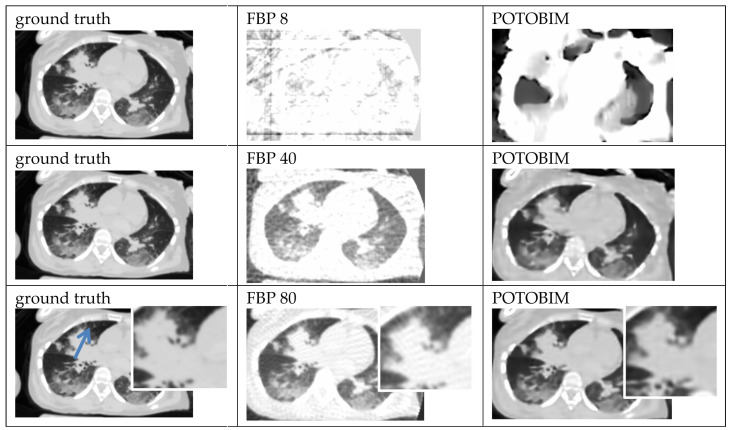
Exemplified images (heart-lung) for reconstruction with 8, 40 and 80 projections used with the FBP (**center**) and POTOBIM (**right**). In comparison, the reference image using all projections (**left**). Detailed view (white box) displaying relevant anatomical region marked by blue arrow.

Examples of the sparse-view reconstructions for 8, 40 and 80 projection angles are shown for dental crown in Figure 2 which can be seen in the left maxilla (blue arrow). Enlargements of this area are used in the last row to show the differences between ground truth, FBP and POTOBIM for 80 projections. Additional examples of sparse-view reconstructions for subdural hematoma, spleen and the transition between heart and lung are shown in Figure A1, Figure A2 and Figure A3. For example, in the case of the left-sided subdural hematoma one can barely see the hematoma at 80 projections for POTOBIM, and it is unclear at 80 projections for FBP.

At 8 projections, a significantly worse image impression was found in all cases compared to the reference image (*p* < 0.001; One-Sample Wilcoxon Signed Rank Test). This is true for both the average values of POTOBIM vs. ground truth and FBP vs. ground truth.

Overall, the image quality is not sufficient for a satisfactory evaluation since the structures can partly not be visualized in both alternatives.

Again, at 40 projections a significantly worse image impression could be demonstrated (*p* < 0.001; One-Sample Wilcoxon Signed Rank Test). This is true for the average values of POTOBIM vs. ground truth as well as FBP vs. ground truth. 

Even at 80 projections there is a significant difference (significantly worse; *p* < 0.001; One-Sample Wilcoxon Signed Rank Test). This again applies for the average values of POTOBIM vs. ground truth as well as FBP vs. ground truth.

The image quality of the foreign bodies (dental crown; total hip endoprosthesis) as well as the boundary between the heart and lung (Figure A3) can be described as comparable for POTOBIM as well as the dental crown (Figure 2) and the right frontal hematoma for FBP. To compare the two methods itself, the demarcation from the surroundings is comparable for both methods; however, the internal structure differs slightly depending on the method used.

It is only at 80 projections that the tomographic reconstruction by means of BNN produces approximately equivalent images in four cases. In the majority (13 cases), the image is qualitatively worse than the reference image. 

As for an intracranial hemorrhage, one can barely or not surely demarcate blood at all; for the evaluation of organs even if the outer structure like a capsule is relatively good to differentiate (for example the spleen; Figure A2), the internal structure is not good to assess.

A subjective slightly or clearly better image impression could be achieved in no single case (this applies for ground truth vs. POTOBIM and ground truth vs. FBP). 

The individual evaluations for the respective regions or structures are summarized in Table 1 and Table 2.

### 3.2. Quantitative Evaluation

**Figure 3:**
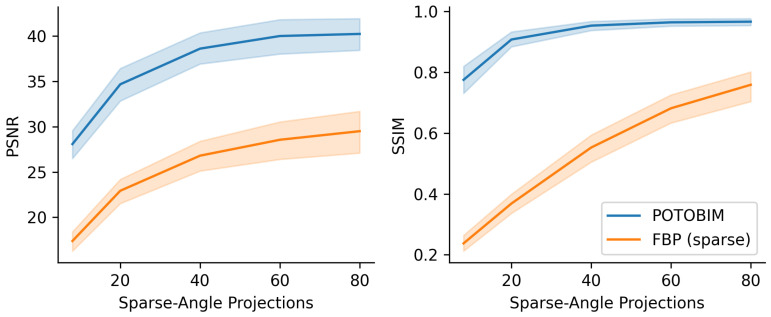
Mean value (line) and standard deviation (shaded) for PSNR (**left**) and SSIM (**right**) over the number of projections used (8–80) for POTOBIM (blue) and FBP (orange).

The quantitative assessment of the sparse-view reconstruction compared to the ground truth is shown in Figure 3. PSNR and SSIM were averaged for the 17 data sets and plotted over the number of simulated projections. The shaded area around the mean corresponds to the standard deviation. Initially, the additional projections suggest a significant increase in image quality as from 8 to 20 projections, the PSNR increases from 28.08 to 34.68 and from 17.38 to 22.93 for POTOBIM and FBP, respectively. The SSIM also increases initially from 0.77 to 0.91 for POTOBIM and from 0.23 to 0.37 for FBP. A further increase to 40 projections leads to an increased PSNR of 38.61 and 26.89 and a SSIM of 0.95 and 0.55 POTOBIM and FBP, respectively. After that, FBP benefits from additional projections with a PSNR of 28.55 and 29.51 and a SSIM of 0.68 and 0.76 for 60 and 80 projections per rotation, respectively. In contrast, the curve flattens out for POTOBIM and the PSNR for 60 and 80 projections yield 39.64 and 40.23, respectively, while the SSIM yields a similarity of 0.96 to the reference image in both cases. 

The average computation time for POTOBIM reconstruction with 1 × 10^5^ iterations was 95 min per slice using a RTX 3090 GPU, Nvidia Corp. (Santa Clara, CA, USA). 

## 4. Discussion and Conclusions

In the field of clinical imaging such as CT, one main goal is to explore methods that apply lower radiation doses than conventional CT and still have good diagnostic qualities. In addition to already established methods such as FBP and IR [11], a new method and its applicability should be explored. Deep learning methods are at this point innovative methods to which the attention should be directed. In general, the development of deep learning methods aims to improve image quality—while a reduction in radiation dose is a positive side effect [11]. 

Established reconstruction methods in the context of deep learning methods are convolutional neural networks (CNNs) trained in a supervised procedure [28]. In the current study, POTOBIM, an unsupervised approach of Bayesian CNNs, was applied as a new method to counter the problem described above. As already shown in the study of Laves et al. [24], POTOBIM is a method that shows a lower reconstruction error in the region of interest compared to other unsupervised methods aiming to prevent hallucinations during reconstruction. 

However, POTOBIM remains inferior to conventional CT in this given study. A slightly or clearly worse image impression was produced when using 8, 40 or 80 projections instead of the original number. 

Given these findings, the POTOBIM method does not yet seem to be suitable for clinical routine in the field of low-dose CT due to very long computing times of more than one hour as well as insufficient qualitative image impressions. However, there were four examples of POTOBIM sparse-view reconstructions with a diagnostic quality comparable to the dense-view ground truth. So, while the general application does not seem ready yet for widespread clinical use, positive results underline the potential of BNNs for low-dose CT.

Other deep learning-based methods are already in clinical use, e.g., TrueFidelity (GE Healthcare) or AiCE (Canon Medial Systems). The development of these reconstruction methods was primarily addressed with improving quality [11] and it remains unclear whether reduced currents or also reduced projections per rotation were used [29,30]. This makes direct comparisons to our results difficult. 

From the basic point of view, deep learning reconstruction methods are well suited to produce better image quality. However, from the evaluation of the current study, it can be said that the POTOBIM method is not yet mature enough to provide qualitatively comparable images in sparse-view CTs. The application of 80 projections in this procedure (POTOBIM) does not reach the level of conventional CT images in terms of quality. The quantitative analysis shows that POTOBIM did not seem to benefit from a higher dose beyond 60 projections per rotation. Thus, the procedure seems suitable for low-dose reconstruction only and needs to be further developed to be competitive.

The results also address the second question raised, as even though the quantitative evaluation shows an increase in image quality, especially with regard to 40 projections, this is not perceived to the same extent by the radiologist expert. An increase in image quality (viewed quantitatively) is therefore not automatically accompanied by sufficient image quality (recorded qualitatively). It has been shown that most objective quality metrics do not also directly translate to diagnostic quality in magnetic resonance images [31]. The results presented here underline similar findings for low-dose CT reconstruction.

The given study has some relevant limitations. Firstly, the number of CT scans included was low for economic reasons and thus, only partially allows generalizable conclusions. Further investigations could consider additional reconstruction algorithms, additional anatomical structures or involve multiple medical experts in the evaluation. Secondly, we used postmortem scans from our daily routine in an institute of legal medicine. The issue of reduced radiation exposure is no longer important for the cadavers themselves but should be addressed here also for the technical staff. Thirdly, sparse-view reconstruction was only tested for simulated sinograms that are highly limited due to the assumption of parallel beams for 2D slices.

In conclusion, our findings underline that future studies on new or improved reconstruction methods should be compared to existing approaches both quantitatively and qualitatively and postmortem centers are excellent study locations. In the best case, a reduction in radiation in sparse-view CTs could be achieved with the same or improved image quality. While we have investigated multiple examples of anatomically relevant structures for our evaluation, other scenarios would also be valuable to consider in future research steps. It would be interesting to study newly developed reconstruction methods with regard to the detection of lesions in organs or for arterial and venous examinations after application of contrast medium. 

## Figures and Tables

**Table 1:** VGA for POTOBIM vs. ground truth after Ludewig et al. [27].

Region/Structure	8×	40×	80×
Femur (both sides)	5	4	4
Heart-lung	5	4	3
Brain tissue	5	5	5
Total hip endoprosthesis left	5	4	3
Total hip endoprosthesis right	5	4	3
Lung	5	5	4
Lumbar vertebral body 5	5	4	4
Rectus abdominis muscle	5	5	4
Dental crone	5	4	3
Spleen	5	5	5
Kidney left	5	5	5
Pancreas	5	5	5
Subcutaneous adipose tissue	5	4	4
Thyroid gland	5	5	4
Hematoma right frontal	5	4	4
Subdural bleeding right	5	5	5
Subdural hematoma left	5	5	5

**Table 2:** VGA for FBP vs. ground truth after Ludewig et al. [27].

Region/Structure	8×	40×	80×
Femur	5	4	4
Heart-lung	5	5	4
Brain tissue	5	5	5
Total hip endoprothesis left	5	4	4
Total hip endoprothesis right	5	4	4
Lung	5	5	4
Lumbar vertebral body 5	5	4	4
M. rectus abdominis	5	5	4
Dental crone	5	4	3
Spleen	5	5	4
Kidney left	5	5	4
Pancreas	5	5	4
Subcutaneous adipose tissue	5	5	4
Thyroid gland	5	5	4
Hematoma right frontal	5	4	3
Subdural bleeding right	5	5	4
Subdural hematoma left	5	5	5

## Data Availability

The data presented in this study are available on reasonable request from the corresponding authors. The data are not publicly available due to ethical and legal restrictions.
